# Nutritional situation among Syrian refugees hosted in Iraq, Jordan, and Lebanon: cross sectional surveys

**DOI:** 10.1186/s13031-016-0093-6

**Published:** 2016-11-16

**Authors:** S. M. Moazzem Hossain, Eva Leidman, James Kingori, Abdullah Al Harun, Oleg O. Bilukha

**Affiliations:** 1 Health and Nutrition, United Nations Children’s Fund, Middle East and North Africa Region, P.O. Box 1551, Amman, 11821 Jordan; 2 Emergency Response and Recovery Branch, Division of Global Health Protection, Center for Global Health, Centers for Disease Control and Prevention, Building 2500, Mailstop E-22, 2500 Century Parkway NE, Atlanta, GA 30345 USA

**Keywords:** Syria, Refugee, Nutrition, Health, Survey

## Abstract

**Background:**

Ongoing armed conflict in Syria has caused large scale displacement. Approximately half of the population of Syria have been displaced including the millions living as refugees in neighboring countries. We sought to assess the health and nutrition of Syrian refugees affected by the conflict.

**Methods:**

Representative cross-sectional surveys of Syrian refugees were conducted between October 2 and November 30, 2013 in Lebanon, April 12 and May 1, 2014 in Jordan, and May 20 and 31, 2013 in Iraq. Surveys in Lebanon were organized in four geographical regions (North, South, Beirut/Mount Lebanon and Bekaa). In Jordan, independent surveys assessed refugees residing in Za’atri refugee camp and refugees residing among host community nationwide. In Iraq, refugees residing in Domiz refugee camp in the Kurdistan region were assessed. Data collected on children aged 6 to 59 months included anthropometric indicators, morbidity and feeding practices. In Jordan and Lebanon, data collection also included hemoglobin concentration for children and non-pregnant women aged 15 to 49 years, anthropometric indicators for both pregnant and non-pregnant women, and household level indicators such as access to safe water and sanitation.

**Results:**

The prevalence of global acute malnutrition among children 6 to 59 months of age was less than 5 % in all samples (range 0.3–4.4 %). Prevalence of acute malnutrition among women 15 to 49 years of age, defined as mid-upper arm circumference less than 23.0 cm, was also relatively low in all surveys (range 3.5–6.5 %). For both children and non-pregnant women, anemia prevalence was highest in Za’atri camp in Jordan (48.4 % and 44.8 %, respectively). Most anemia was mild or moderate; prevalence of severe anemia was less than or equal to 1.1 % in all samples of children and women.

**Conclusions:**

Despite the ongoing conflict, results from all surveys indicate that global acute malnutrition is relatively low in the assessed Syrian refugee populations. However, prevalence of anemia suggests a serious public health problem among women and children, especially in Za’atri camp. Based on these findings, nutrition partners in the region have reprioritized response interventions, focusing on activities to address micronutrient deficiencies such as food fortification.

**Electronic supplementary material:**

The online version of this article (doi:10.1186/s13031-016-0093-6) contains supplementary material, which is available to authorized users.

## Background

Armed conflict has spread throughout Syria, with warring parties, including government forces, non-state armed groups, and terrorist groups, fighting to expand their areas of influence and gain control of strategic roads, settlements and critical resources. In the sixth year of the conflict, violence in Syria has “neither abated nor diminished in brutality,” according to the United Nations Security Council [1, 2].

The humanitarian consequences of this conflict are dramatic. According to United Nations (UN) and regional partners, Syria is the largest displacement crisis globally [3]. Entering the fourth year of the conflict, around the time of our assessments, an estimated 8.8 million people had been displaced, almost half the population of Syria [4]. Of them, more than a quarter have been forced to cross international borders and seek refuge in neighboring countries [4]. An estimated one in five registered refugees are children under 5 years of age [5].

Previous complex emergencies have demonstrated that conflict and displacement often result in a deterioration of the nutritional status of populations. In the context of prolonged conflict, affected populations often become dependent on humanitarian assistance for food and as a result experience reduced dietary diversity and meal frequency [6–8]. Separation of children from caretakers as a result of the conflict and displacement, also often affects infant and young child feeding (IYCF) practices [9, 10]. Deterioration of water, sanitation, and health infrastructure, as well as access to key services can compound the effects of crisis on nutrition [11].

Potential for nutrition insecurity was of particular concern in Syria as assessments of the nutritional status of children prior to the conflict suggested some baseline deficiencies. According to the 2009 Syrian Family Health Survey (SFHS) and the 2006 Multiple Indicator Cluster Survey (MICS), levels of total wasting in Syria were greater than 8 %, poor by World Health Organization (WHO) Crisis Classifications [12–14]. Levels of stunting were greater than 20 % in both assessments.

The scale of the displacement from Syria has further contributed to concerns about the nutritional status of displaced persons. Of the more than two million Syrian refugees displaced in 2013-14, the majority were hosted in Lebanon, Jordan, Turkey, and Iraq [4]. The displacement, in absolute terms and relative to the host population, has overwhelmed some of these neighboring countries. For example, Lebanon, a country of just over four million people, registered its millionth Syrian refugee shortly after the survey in Lebanon concluded [15].

While nutrition partners cautioned of a deteriorating situation in Syria, limited data existed at the time of our surveys. Better information was needed to inform the ongoing response—estimated at $30 million in nutrition programming and $1.1 billion in food and agriculture activities for 2014 [16]. This need prompted calls for rigorous and representative assessments to confirm the nutritional situation of Syrian refugees [17].

In this context, nutrition surveys were organized in Jordan, Lebanon and Iraq to generate evidence as to the nutritional status of Syrian refugees with a primary objective of assessing the level of acute malnutrition among women and children. Additional indicators including those related to anemia, access to health care, morbidity, IYCF practices, and water and sanitation helped contextualize these findings. Surveys in Lebanon, Jordan and Iraq also allowed us to compare whether the nutritional status of refugees depended on the country where refugees settled or the type of settlement (in refugee camps or integrated with host population).

## Methods

### Study design and sample size

Data used in this analysis were obtained from cross-sectional surveys of Syrian refugees from seven sites in three countries: Lebanon, Jordan and Iraq. Data were obtained from the United Nations Children’s Fund (UNICEF) in Lebanon and Iraq and the Office of the United Nations High Commissioner for Refugees (UNHCR) in Jordan. In Lebanon, independent survey samples were selected in the four geographical regions of the country: North, South, Beirut/Mount Lebanon and Bekaa. Since there were no established refugee camps in Lebanon, all surveyed refugees resided in host communities. In Jordan, two independent surveys were conducted: among refugees residing in Za’atri refugee camp and among refugees residing among host communities (outside of camps) nationwide. In Iraq, the survey was conducted among refugees residing in Domiz refugee camp in the Kurdistan region of Iraq. Data collection took place between October 2 and November 30, 2013 in Lebanon, April 12 and May 1, 2014 in Jordan and May 20 and 31, 2013 in Iraq.

For surveys in Jordan and Lebanon, sample sizes were calculated based on estimated prevalence of global acute malnutrition, required precision, and design effects which were informed by results of previous cross-sectional nutrition surveys—a September 2012 survey in Lebanon and a November 2012 survey in Jordan [18, 19]. Adjusting for non-response rate and household demographics, 340, 270, 330 and 260 households were randomly selected in the North, South, Beirut/Mount Lebanon and Bekaa regions of Lebanon, respectively. Similarly in Jordan, 337 and 515 households were selected in Za’atari Camp and in host community, respectively. Parameters used for estimating sample size in Lebanon and Jordan are provided in Additional file 1. In Iraq, a 30 cluster by 30 children survey design was used [20].

A list of registered refugees and persons awaiting registration maintained by the UNHCR was used as a sampling frame for all surveys, since this database included the most updated information on recently arrived refugees. In all surveys, the UNHCR database was used to select clusters randomly, proportional to the number of refugees residing in each administrative area or camp section. In non-camp settings, households within each cluster were randomly selected from the UNHCR database and called in advance of the field visit to confirm their availability, willingness to participate, and current address. In Jordan, 83 % of households in the database had functional phone numbers. In both of the refugee camps (Domiz in Iraq and Za’atari in Jordan), households were systematically selected; sampling intervals were calculated using household listings by block obtained from community leaders or local health volunteers.

### Measurements

The information on household, children and women was collected by teams that included both nationals and Syrian refugees. Teams composed of at least three members each (an interviewer, anthropometry measurer(s), and a hemoglobin (Hb) technician where Hb was measured) received between 2 and 7 days of training, including a field test. All surveys collected data on basic demographics, anthropometry, recent illness, breastfeeding practices, and household level indicators related to water and sanitation. The surveys in Jordan and Lebanon also collected data on Hb concentration, food security and access to antenatal care (ANC). Household level indicators were excluded from the analysis of Iraq data, as the Domiz sample only included households with children under 5 years of age, and therefore was not a representative sample of all households in the camp.

Hb concentration was measured in children aged 6 to 59 months and non-pregnant women of reproductive age (15–49 years). Pregnancy status was self-reported. Hemoglobin was measured in capillary blood using a HemoCue Hb 301+ photometer following a standard procedure [21]. Anemia in children was classified according to WHO definitions as severe (hemoglobin < 7 g/dL), moderate (7 g/dL ≤ hemoglobin < 10 g/dL), or mild (10 g/dL ≤ hemoglobin < 11 g/dL) [22]. In non-pregnant women, anemia was classified as severe (hemoglobin < 8 g/dL), moderate (8 g/dL ≤ hemoglobin < 11 g/dL), or mild (11 g/dL ≤ hemoglobin < 12 g/dL) [22].

Anthropometry indicators in children aged 6 to 59 months were measured following standard procedures [23]. Weight was measured with a digital Seca scale, accurate to 0.1 kg. Height or recumbent length was measured with a wooden ShorrBoard, accurate to 1 mm. Children’s age was ascertained from UNHCR registration cards or, if unavailable, from immunization cards, birth certificates, or caregiver recall. Presence of bilateral pitting edema was assessed by survey teams; all suspect cases were verified by a supervisor. Acute malnutrition (based on weight-for-height z-score and the presence of edema), stunting (based on height-for-age z-score), and underweight (based on weight-for-age z-score) were defined and classified as moderate (–3 ≤ z-score < –2) and severe (z-score < –3) according to WHO definitions [24]. Extreme outliers, defined using a flexible exclusion range criterion (±4 z-scores from the observed mean) were excluded from the analysis [25, 26]. Surveys assessed nutritional status of all women of reproductive age (15 to 49 years), classifying women with a mid-upper arm circumference (MUAC) less than 23.0 cm as malnourished [27].

Additional indicators were ascertained by self-report. Diarrhea was defined as three or more loose or watery stools in a 24-h period during the preceding 2 weeks. Current breastfeeding included children put to breast during the previous 24-h period. In Lebanon and Iraq both indicators were assessed among all children 0–59 months; in Jordan children 0–5 months were not assessed for diarrhea. Access to antenatal care and iron-folic acid supplements was assessed in all pregnant women (15–49 years). A refugee household living outside of a refugee camp was considered to be hosted if they were residing in the same dwelling as citizens of the host country at the time of the assessment. Survey instruments were modified in each country to meet the information needs of the ongoing response; indicators not collected consistently across settings were excluded.

In Jordan, data were collected on mobile phones with an Android OS and uploaded daily. Anthropometric measurements were recorded on both the phone and paper allowing supervisors to verify data entry. In Lebanon and Iraq, all data were recorded on paper questionnaires.

### Statistical analysis

For all surveys, ENA for SMART software 2011 (Version: Aug. 4, 2014) [28] was used to generate standardized z-scores based on the 2006 WHO Growth Standards and analyze anthropometry data [24]. Additional variables were analyzed with EPI INFO 3.5.3 software, SPSS version 17.0, and STATA/IC 13.1. Ninety-five percent confidence intervals were calculated adjusting for the complex survey design. Significance was tested adjusting for clustering using the procedure described by Donner et al. [29] A *p*-value <0.05 was considered statistically significant.

## Results

### Survey samples

Final sample in Jordan included 329 households in Za’atari (response rate of 97.6 %) and 504 households in the host community (response rate of 97.9 %). In Lebanon, 326 (response rate of 95.9 %), 264 (response rate of 97.8 %), 327 (response rate of 99.1 %) and 255 (response rate of 98.1 %) households were assessed in North, South, Beirut/Mount Lebanon and Bekaa, respectively. These households included an average of 1.1 children aged 0–59 months per household in Jordan and an average of 1.3 children aged 0–59 months per household in Lebanon. In Iraq, 944 children 0–59 months were included. The number and demographics of children in each sample are presented in Table 1.

**Table 1:** Survey sample characteristics, Syrian refugees in Jordan, Lebanon and Iraq, 2013-2014

	Jordan 2014		Lebanon 2013				Iraq 2013
Characteristic	Za’atri (Refugee Camp)	National (Outside Camp)	North	South	Beirut	Bekaa	Domiz (Refugee Camp)
Number of Households in Sample	329	504	326	264	327	255	--
Number of Children (0–59 mo) in Sample	355	541	409	329	465	332	944
Age (mo) --number (%) of survey sample							
0–5	28 (7.9)	58 (10.7)	31 (7.6)	19 (5.8)	42 (9.0)	24 (7.2)	104 (11.0)
6–23	115 (32.4)	166 (30.7)	132 (32.3)	109 (33.1)	127 (27.3)	103 (31.0)	488 (51.7)
24–59	212 (59.7)	317 (58.6)	236 (57.7)	196 (59.6)	291 (62.6)	203 (61.1)	352 (37.3)
Male Sex	185 (52.1)	279 (51.6)	182 (44.5)	144 (43.8)	241 (51.8)	166 (50.0)	466 (49.4)
Median age – mo	29	29	28	31	29	29	26
Number of Women (15–49 years) number (%) of survey sample	359	704	567	437	527	405	
Non-pregnant	314 (87.5)	630 (89.5)	503 (88.7)	378 (86.5)	470 (89.2)	354 (87.4)	--
Pregnant	45 (12.5)	74 (10.5)	64 (11.3)	59 (13.5)	57 (10.8)	51 (12.6)	--

Women aged 15–49 years were assessed in surveys conducted in Jordan and Lebanon. The samples in Jordan included 359 women (of them, 12.5 % pregnant) in Za’atri and 704 women (10.5 % pregnant). In Lebanon, survey samples included 567 women (11.3 % pregnant) in the North, 437 women (13.5 % pregnant) in the South, 527 women (10.8 % pregnant) in Beirut/Mount Lebanon, and 405 women (12.6 % pregnant) in Bekaa (Table 1).

### Anthropometry

Findings suggest that the prevalence of global acute malnutrition (GAM) among children was low among Syrian refugees across the region, less than 5 % in all samples (range 0.3–4.4 %) (Table 2). In all samples in Jordan and Lebanon the means of weight-for-height (WHZ) z-score were higher than zero (range 0.23–0.39), indicating that the populations were slightly overweight compared to the WHO standard population. Mean WHZ z-score in Domiz was approximately zero (-0.02).

**Table 2:** Prevalence of malnutrition and mean anthropometry scores among children 6–59 months of age, Syrian refugees in Jordan, Lebanon and Iraq, 2013–2014

	Jordan 2014		Lebanon 2013				Iraq 2013
	Za’atri (Refugee Camp)	National (Outside Camp)	North	South	Beirut	Bekaa	Domiz (Refugee Camp)
Global acute malnutrition, children aged 6–59 mo							
Global – % (95 % CI)	1.2 (0.5–3.2)	0.8 (0.3–2.2)	3.9 (2.5–6.1)	0.3 (0.0–2.6)	0.7 (0.2–2.3)	4.4 (2.3–8.2)	4.1 (2.8–6.1)
Severe – % (95 % CI)	0.3 (0–2.4)	0 (0)	0.3 (0–2.1)	0 (0)	0.2 (0–1.9)	1.7 (0.5–5.4)	1.1 (0.5–2.4)
Mean Z-score (SD)	0.26 (0.90)	0.23 (0.94)	0.29 (0.94)	0.39 (0.93)	0.34 (0.92)	0.29 (0.97)	-0.02 (1.08)
Stunting, children aged 6–59 mo							
Global –% (95 % CI)	16.7 (11.6–23.4)	10.5 (7.6–14.4)	20.1 (15.6–25.4)	21.0 (16.6–26.2)	14.1 (10.8–18.2)	21.0 (16.0–27.2)	19.0 (15.8–22.7)
Severe –% (95 % CI)	3.1 (1.6–5.9)	2.5 (1.4–4.6)	2.8 (1.6–5.0)	4.7 (2.6–8.1)	1.0 (0.4–2.5)	4.5 (2.6–7.8)	5.2 (3.4–7.8)
Mean Z-score (SD)	-0.77 (1.33)	-0.59 (1.15)	-0.99 (1.20)	-1.06 (1.17)	-0.90 (1.03)	-1.07 (1.23)	-0.76 (1.46)
Underweight, children aged 6–59 mo							
Global –% (95 % CI)	4.0 (2.4–6.7)	2.9 (1.7–4.9)	3.9 (2.2–6.8)	4.0 (2.4–6.6)	2.5 (1.2–5.1)	2.8 (1.2–6.0)	6.3 (4.6–8.7)
Severe –% (95 % CI)	0.3 (0–2.3)	0.4 (0.1–1.7)	1.1 (0.4–2.8)	1.0 (0.3–3.0)	0.5 (0.1–2.0)	1.0 (0.3–3.2)	1.4 (0.8–2.4)
Mean Z-score (SD)	-0.24 (1.04)	-0.13 (1.01)	-0.33 (1.04)	-0.29 (1.01)	-0.24 (0.97)	-0.37 (0.99)	-0.39 (1.16)

Prevalence of chronic malnutrition (stunting) was lower among refugee children in the host population in Jordan (10.5 %) compared to other surveys (range 14.1–21.0 %; *p* < 0.05 compared to Iraq Domiz, Lebanon Bekaa, North and South). Mean height-for-age (HAZ) z-score ranged from -0.59 to -1.07 (Table 2).

Prevalence of acute malnutrition based on MUAC among women 15–49 is presented in Table 3. As with acute malnutrition in children, there was little variability between surveys in the prevalence of low MUAC among women, and the prevalence was relatively low in all surveys (range 3.5–6.5 %).

**Table 3:** Anemia prevalence and access to antenatal care in women 15–49 years of age, Syrian refugees in Jordan and Lebanon, 2013–2014^a^

	Jordan 2014		Lebanon 2013			
	Za’atri (Refugee Camp)	National (Outside Camp)	North	South	Beirut	Bekaa
Mid-upper arm circumference (<23 cm) (All Women 15–49 yrs.)						
Total (15–49 years)	5.1 (4.7–5.6)	3.5 (2.4–4.5)	5.3 (3.7–7.4)	6.5 (4.5–9.3)	4.4 (2.9–6.6)	3.7 (2.2–6.0)
Anemia (Non-pregnant women 15–49 yrs.)						
Total anemia (Hb < 12.0 g/dL)	44.8 (38.5–51.0)	31.1 (27.2–35.0)	27.7 (22.3–33.2)	27.0 (20.0–34.2)	29.3 (24.0 –34.6)	18.4 (12.6–24.1)
Mild anemia (Hb < 11.0–11.9 g/dL)	21.2 (16.7–25.7)	17.6 (14.6–20.7)	13.6 (8.7–18.5)	14.0 (7.4–20.7)	16.2 (12.0–20.3)	10.1 (5.2–15.0)
Moderate anemia (Hb < 8.0–10.9 g/dL)	22.5 (17.5–27.5)	12.9 (10.7–15.2)	14.1 (9.8–18.4)	11.8 (7.7–16)	12.2 (8.1–16.3)	7.6 (3.8–11.4)
Severe anemia (Hb < 8.0 g/dL)	1.0 (0–2.4)	0.5 (0–1.0)	0	1.1 (0–2.7)	0.9 (0–2.1)	0.6 (0–2.0)
Hb (g/L) (Non-pregnant women 15–49 yrs.) Mean +/- SD						
Total (15–49 y)	12.0 (1.51)	12.6 (1.48)	12.6 (1.36)	12.6 (1.53)	12.5 (1.65)	13.3 (1.57)
Access to antenatal health care (Pregnant women 15–49 yrs.)						
Currently enrolled in ANC	53.3 (35.2–71.4)	29.7 (16.2–43.3)	50.8 (36.5–65.1)	52.5 (37.5–67.6)	44.4 (31.2–57.7)	45.1 (26.1–64.1)
Received iron-folic acid pill	37.8 (22.9–52.3)	42.5 (29.7–55.2)	33.9 (18.9–48.9)	32.8 (20.2–45.3)	37.7 (19.9–55.5)	44.0 (28.9–60.3)

*Hb* hemoglobin

^a^Data are presented as prevalence (95 % CI) unless noted otherwise

### Anemia

Anemia prevalence in children and non-pregnant women is presented in Tables 3 and 4, respectively. Anemia prevalence was higher in Za’atri camp compared to the other six surveys (*p* < 0.05 for all); in both children and women, total anemia in Za’atri camp exceeded 40 % (48.4 % and 44.8 %, respectively). Prevalence of total anemia among children in the non-camp refugee populations in Jordan (26.1 %) was comparable to the prevalence among children in Lebanon (range 13.9 to 25.8 %) (*p* > 0.05 except for Bekaa). Similarly, the prevalence of anemia among women in the non-camp refugee populations in Jordan (31.1 %) was comparable to the prevalence among women in Lebanon (range 18.4 to 29.3 %) (*p* > 0.05 except for Bekaa).

**Table 4:** Anemia prevalence in children 6–59 months of age and mean hemoglobin levels, Syrian refugees in Jordan and Lebanon, 2013–2014^a^

	Jordan 2014		Lebanon 2013			
	Za’atri (Refugee Camp)	National (Outside Camp)	North	South	Beirut	Bekaa
Anemia (6–59 mo)						
Total anemia (Hb < 11.0 g/dL)	48.4 (42.0–54.9)	26.1 (21.3–30.8)	25.8 (15.9–34.7)	23.4 (16.3–30.6)	21.2 (15.9–27.7)	13.9 (8.2–19.6)
Mild anemia (Hb 10.0–10.9 g/dL)	27.7 (23.0–23.3)	18.5 (14.8–22.2)	20.0 (18.8–28.2)	17.2 (11.0–23.5)	16.3 (11.3–21.2)	12.7 (7.6–17.7)
Moderate anemia (Hb 7.0–9.9 g/dL)	20.4 (16.3–24.5)	7.6 (5.0–10.1)	5.8 (2.8–8.8)	6.2 (1.5–10.9)	4.9 (1.8–8.0)	1.3 (0–3.1)
Severe anemia (Hb < 7.0 g/dL)	0.3 (0–1.0)	0	0	0	0	0
Anemia (6–23 mo)						
Total anemia (Hb < 11.0 g/dL)	64.0 (54.0–74.0)	36.6 (28.9–44.3)	42.9 (30.3–55.4)	30.8 (17.6–44.0)	27.7 (17.9–37.5)	24.1 (14.1–34.2)
Moderate or Severe (Hb <10.0 g/dL)	29.7 (23.1–37.4)	12.2 (7.5–19.1)	9.5 (2.3–16.8)	13.5 (2.8–24.1)	6.2 (2.4–15.2)	1.7 (0–5.4)
Anemia (24–59 mo)						
Total anemia (Hb < 11.0 g/dL)	40.1 (31.9–48.3)	20.5 (15.0–26.0)	14.1 (6–22.2)	19.4 (11.5–27.2)	18.1 (12.4–23.8)	8.2 (2.4–14.0)
Moderate or Severe (Hb <10.0 g/dL)	15.9 (11.4–22.3)	5.1 (3.3–7.9)	3.3 (0–6.8)	2.2 (0–5.2)	4.3 (1.3–7.4)	1.0 (0–3.2)
Hb (g/L) Mean ± SD						
Total (6–59 mo)	11.0 (1.24)	11.7 (1.32)	11.7 (1.16)	11.7 (1.11)	11.8 (1.11)	12.2 (1.13)
6–23 mo	10.5 (1.12)	11.4 (1.27)	11.4 (1.04)	11.5 (1.30)	11.6 (1.04)	11.8 (1.16)
24–59 mo	11.2 (1.23)	12.0 (1.30)	12.0 (1.11)	11.9 (0.96)	11.9 (1.12)	12.5 (1.05)

*Hb* hemoglobin

^a^Data are presented as prevalence (95 % CI) unless noted otherwise

Only one child in 6 surveys was classified as having severe anemia. Prevalence of severe anemia was less than or equal to 1.1 % in all samples of non-pregnant women. The mean hemoglobin level was equal to or greater than 11.0 g/dL for all samples of children and 12.0 g/dL for all samples of women.

The prevalence of total anemia was higher among children aged 6–23 months compared to children aged 24–59 months in all samples (*p* < 0.05 except for Beirut and South). In North and Bekaa Lebanon the prevalence of total anemia among younger children was nearly three times that of older children (42.9 % and 14.1 % in North, 24.1 % and 8.2 % in Bekaa, respectively). Mean hemoglobin ranged from 10.5 to 11.8 g/dL among younger children (6–23 months) and 11.2 to 12.5 g/dL for older children (24–59 months). The prevalence of anemia among non-pregnant women was similar to that in children (*p* > 0.05 in all surveys except Beirut).

### Antenatal care

29.7 % of pregnant women living among the host population in Jordan reported being enrolled in antenatal care, a lower proportion than 53.3 % enrollment reported in the Za’atri camp in Jordan (*p* < 0.05). Enrollment in Za’atri camp was similar to that reported in Lebanon surveys (range 44.4–52.5 %, *p* > 0.05 for all). Enrollment in antenatal care did not exceed 55 % in any of the surveys (Table 3).

The proportion of pregnant women receiving iron-folic pills ranged from 32.8 % in South Lebanon to 44.0 % in Bekaa. The number of women receiving iron-folic supplements represented a subset of women enrolled in antenatal care in all samples except in the host population in Jordan. In this sample, more women received iron-folic than reported enrollment in ANC (Table 3).

### Child morbidity and breastfeeding

The reported 2-week cumulative incidence of diarrhea was highest in North Lebanon (33.3 %) and the two refugee camps, Domiz (32.8 %) and Za’atri (27.0 %). The cumulative incidence in Za’atri was nearly twice that of refugees living among the host population in Jordan (*p* < 0.05). The cumulative incidence of diarrhea was consistently higher among younger children (0–23 months in Lebanon, 6–23 months in Jordan) than older children (24–59 months) (*p* < 0.05 except for Lebanon North and Iraq Domiz) (Table 5).

**Table 5:** Health and nutrition of children aged 0–23 months, by location

	Jordan 2014		Lebanon 2013				Iraq 2013
	Za’atri (Refugee Camp)	National (Outside Camp)	North	South	Beirut	Bekaa	Domiz (Refugee Camp)
Two week prevalence of diarrhea − % (95 % CI)							
Total (0–59 mo)	27.0 (21.1–33.9)^a^	14.5 (11–18.8)^a^	33.3 (26.4–41.1)	23.6 (18.3–29.9)	17.2 (12.3–23.5)	26.7 (21.0–33.3)	32.8 (25.6–40.9)
0–23 mo	39.1 (29.6–49.6)^a^	21.1 (14.9–28.9)^a^	38.0 (28.7–48.4)	36.7 (29.8–44.2)	25.0 (17.5–34.3)	39.4 (32.3–46.9)	37.5 (29.4–46.4)
24–59 mo	20.4 (14.2–28.3)	11.0 (7.9–15.3)	30.1 (23.0–38.3)	15.0 (9.5–22.7)	12.7 (8.0–19.7)	18.7 (12.4–27.3)	29.4 (22.1–37.9)
Current breastfeeding practice − % (95 % CI)							
Total (0–23 mo)	66.7 (57.6–74.7)	68.1 (59.8–75.3)	57.8 (47.7–67.3)	62.0 (49.8–72.8)	65.6 (56.2–74.0)	60.0 (47.2–71.6)	44.5 (40.2–48.9)
0–5 mo	100	94.4 (77.8–98.8)	88.0 (71.8–95.5)	93.8 (57.3–99.4)	93.1 (73.3–98.5)	84.6 (46.5–97.2)	78.6 (68.7–86.1)
6–11 mo	97.4 (82.0–99.7)	86.4 (73.1–93.7)	61.3 (40.7–78.5)	70.8 (51.9–84.6)	80.0 (61.7–90.9)	71.4 (48.0–87.1)	58.2 (48.6–67.2)
12–23 mo	24.5 (14.2–39.0)	44.1 (32.1–56.9)	43.3 (29.9–57.8)	48.1 (30.8–65.8)	45.3 (35.1–56.0)	46.3 (30.9–62.5)	25.1 (20.2–30.7)

^a^Prevalence of diarrhea in Jordan was not assessed among children 0–5 months of age

Current breastfeeding was assessed for all children aged 0–23 months. Prevalence was highest among the youngest cohort (children aged 0–5 months) and declined after 5 months of age in all samples. Current breastfeeding among children under two was lower in Domiz camp (44.5 %) compared to other surveys (range 57.8–68.1 %, *p* < 0.05 for all). In both camps, the proportion of currently breastfed children declined sharply after 1 year of age, to 24.5 % of children aged 12–23 months in Za’atri and to 25.1 % of children aged 12–23 months in Domiz (Table 5).

### Water, sanitation, and accommodations

Main source of drinking water, a proxy for access to safe drinking water, is presented in Table 6. The main source of drinking water in Za’atri camp was shared water tanks (sourced by tanker trucks), reported by 76.9 % of refugees. For refugees living among host populations in Jordan and Lebanon municipal tap water and bottled water were the primary source; tanked water was an uncommon source, reported to be the primary source of water by less than 2 % households. Bottled water was more common than tap water among refugees residing out of camp in Jordan (56.5 % and 38.4 %, respectively) and in Beirut/Mount Lebanon (64.0 % and 26.8 % respectively); the reverse was true in North, South and Bekaa regions of Lebanon. Boreholes were another notable source of water in Lebanon, reported to be the main source of water for 16.3 % of households in Bekaa and 20.4 % in North Lebanon.

**Table 6:** Water, sanitation, and accommodations of refugee households, Syrian refugees in Jordan and Lebanon, 2013–2014

	Jordan 2014		Lebanon 2013			
	Za’atri (Refugee Camp)	National (Outside Camp)	North	South	Beirut	Bekaa
Main source of drinking water − % (95 % CI)						
Running Water (Tap)	10.5 (46–22.1)	38.4 (29.7–47.9)	44.4 (35.1–54.2)	54.4 (44.2–64.2)	26.8 (17.9–38.0)	44.3 (36.0–52.9)
Bottled (purchased) water	12.6 (7.9–19.5)	56.5 (46.7–65.8)	14.8 (10.7–21.6)	31.9 (23.1–42.3)	64.0 (52.5–74.1)	19.4 (11.2–31.4)
Water tank or tanker truck	76.9 (64.6–85.9)	2.0 (0.9–4.5)	0	0	0	0.4 (0–3.1)
Other	0	3.2 (1.7–6)	40.7 (30.9–51.4)	13.7 (9.4–19.4)	9.2 (5.6–14.9)	36.0 (29.1–43.5)
Primary toilet used by household − % (95 % CI)						
Public Toilet	31.1 (20.6–43.9)	0.2 (0–1.5)	1.5 (0.4–5.7)	0.4 (0–29.9)	1.5 (0.7–3.6)	2.0 (0.7–5.4)
Private - Not Shared	38.2 (30.0–47.0)	56.3 (50.9–61.5)	68.2 (59.8–75.6)	72.7 (64.3–79.7)	71.1 (62.7–78.2)	64.7 (56.8–71.8)
Private - Shared with other household(s)	18.5 (13.2–25.2)	42.7 (37.7–48.0)	29.6 (22.1–38.5)	26.2 (19.4–34.3)	27.1 (19.9–35.7)	32.5 (24.9–41.2)
Other/Unknown	12.3 (6.0–23.7)	0.8 (0.3–2.1)	0.6 (0–4.6)	0.8 (0.2–3.2)	0.3 (0–2.3)	0.8 (0.1–5.9)
Sharing Accommodation − % (95 % CI)						
Not Sharing Accommodations	--	66.0 (56.9–74.1)	64.9 (56.4–72.6)	67.1 (59.6–73.7)	59.5 (51.7–66.8)	68.9 (61.3–75.6)
Hosted - Sharing with a national	--	2.3 (1.1–5.1)	13.2 (7.9–21.3)	8.3 (4.5–15.0)	13.2 (9.1–18.8)	12.6 (8.2–18.9)
Not Hosted - Sharing w/ 1 refugee HH	--	20.7 (15.2–27.6)	12.3 (8.2–18.1)	14.8 (10.3–20.8)	15.7 (10.8–22.1)	9.8 (6.3–15.0)
Not Hosted - Sharing w/ >1 refugee HH	--	10.9 (7.3–16.0)	9.5 (6.3–14.1)	9.9 (6.2–15.4)	11.7 (7.6–17.5)	8.7 (5.3–13.8)

The majority of households in all samples had access to private toilets, including refugees in Za’atri refugee camp. Within Za’atri camp, less than a third (31.1 %) of the population relied on public toilet blocks or toilets in public places like markets or clinics. Private, individual or shared toilets were reported to be more common, used by 38.2 % and 18.5 % of households, respectively. For refugees living among the host population, the majority (range 56.3 % to 72.7 %) of household had at least one private toilet per household. Private toilets were often shared in dwellings shared by more than one household.

The majority of refugee households in Lebanon and Jordan residing outside of a refugee camp were not sharing accommodations with either host nationals or other refugees (range 59.5 % to 68.9 %). Cohabiting with host nationals was less common in Jordan (2.3 % of households) compared to samples in Lebanon (range 8.3 % to 13.2 %). Refugees residing in host community were more likely to be cohabiting with other refugees than with host nationals.

## Discussion

Data presented here suggest that despite the magnitude of the crisis, Syrian refugees have not experienced elevated rates of acute malnutrition. Among both women and children, the prevalence of acute malnutrition was relatively low. Global acute malnutrition prevalence among children aged 6 to 59 months was less than the WHO threshold of “acceptable” (<5 %) in all settings [13]. Prevalence of acute malnutrition among women, as measured by mid-upper arm circumference, also indicated relatively low levels of wasting (range 3.5–6.5 %).

Taken together, these data provide a more comprehensive picture of acute malnutrition among Syrian refugees affected by the conflict. Data on the nutrition status of displaced persons within Syria remains sparse given security and access restraints. One of the few representative surveys, a survey of children 6 to 59 months of age in Idleb governorate in June 2014, found that the prevalence of global acute malnutrition was 1.1 %, consistent with the low prevalence we observed among refugee populations [30]. The levels of acute malnutrition observed, however are notably lower than the prevalence reported in pre-conflict national assessments of children in Syria—9.3 % according to the 2009 SFHS and 8.6 % according to the 2006 MICS [12, 14]. The prevalence estimates observed in our assessments are more similar to national estimates from neighboring countries now hosting refugees. Demographic Health Survey (DHS) data from Jordan in 2012 estimated a prevalence of 2.4 % for total wasting and 0.6 % for severe wasting [31]. A 2010 national micronutrient survey in Jordan similarly found the prevalence of total wasting among children 12 to 59 months to be 3.5 % [32]. Estimated prevalence among long-term Palestinian refugees in Lebanon in 2011 also suggest wasting to be below crisis levels (4.7 %) [33]. Prevalence of wasting in Iraq documented in the 2011 MICS was slightly higher (7.4 %) [34]. These findings together suggest that at the time of the assessments, contrary to media reports and popular opinion, Syrian refugee children were not experiencing crisis levels of acute malnutrition but rather had levels comparable to the host populations among which they were displaced.

Whereas for acute malnutrition a change in prevalence from pre-crisis was observed, levels of stunting among refugees in most of our assessments were similar to estimates of stunting in Syria pre-crisis. This distinction is consistent with the fact that stunting is a measure of chronic malnutrition and is less affected by acute changes in food security. Stunting was estimated to be 23.0 % in the 2009 SFHS and 22.4 % in the 2006 MICS [12, 14]. These pre-crisis levels are very similar to that estimated in North, South and Bekaa regions of Lebanon (range 20.1–21.0 %), and not markedly different from stunting prevalence in Za’atri refugee camp (16.7 %). Interestingly, the prevalence of stunting in our assessment of non-camp based refugees in Jordan was lower than in other contexts surveyed (10.5 %); this prevalence is more similar to the prevalence of stunting estimated in Jordan in the 2012 DHS (7.7 %) than estimates from Syria pre-conflict [31]. Prevalence of stunting in our assessment of refugees in Beirut (14.1 %) was more similar to that estimated among Palestinian refugees in Lebanon (13.3 %) [33]. This may reflect a degree of integration of non-camp refugees with the host populations in Jordan and Beirut, Lebanon.

Levels of anemia recorded were high in all assessed settings, but only a problem of major public health significance in the Za’atari refugee camp in Jordan, according to WHO classification [22]. In Za’atari, prevalence of anemia was 48.4 % among children aged 6 to 59 months and 44.8 % among non-pregnant women. High prevalence of anemia among refugees has been documented in many camp-based refugee populations. A study of Bhutanese refugee children in Nepal documented a prevalence of 43.3 % in 2007, prior to introduction of micronutrient supplementation [35]. A study of refugees in long-term African refugee camps found even higher prevalence estimates among children in camps in Kenya (61.3 %), Uganda (72.9 %), and Ethiopia (62.9 %) [8]. High prevalence of anemia among refugees has been attributed in part to the reliance on food rations in camps, an explanation consistent with our finding that prevalence among the camp based refugees in Za’atri was higher than in non-camp based refugees in Jordan and in Lebanon [7, 8, 35]. In our assessments of non-camp based refugee populations, anemia levels were more consistent with the host populations. Among refugee children in Jordan living in the host population, total anemia prevalence in our survey was slightly lower (26.1 %) than that documented in the Jordanian population among children under five in the 2012 Jordan DHS (32.4 %) [31]. Similarly, the prevalence of anemia among refugee women residing in host population in Jordan in our survey (31.3 %) was also just below the estimated prevalence from the 2012 Jordan DHS (33.5 %).

For both women and children, prevalence of severe anemia was low, 1.1 % or less in all surveys. In Lebanon, no cases of severe anemia were identified among children. Supplementation with micronutrient powder has been shown in a randomized trial to have no significant effect on hemoglobin levels among children with hemoglobin levels ≥10.0 g/dL [36]. These findings have subsequently been confirmed in studies of Bhutanese refugee children [35]. As such, micronutrient powder supplementation may have negligible benefits in populations with a low prevalence of children with hemoglobin <10.0 g/dL, as is the case among refugees in Lebanon and Jordan. Instead, interventions that help ensure a more diversified, micronutrient rich diet may better address the micronutrient deficiencies in the refugee populations than powders, particularly given the rich diversity of food available in markets outside the camps.

Self-reported data provided additional information to contextualize the nutrition findings. With respect to antenatal care, all six assessments in Jordan and Lebanon found that fewer than 55 % of pregnant women 15–49 years of age were enrolled in ANC. Pre-crisis, Syria reported high utilization of reproductive health services. A reported 85.3 % of pregnant women received ANC services one or more times during pregnancy in Syria; 93.0 % of deliveries were attended by a skilled professional [12]. Declines in antenatal care coverage may in part be related to cost. Antenatal care in Syria was free or inexpensive relative to services in Jordan and Lebanon [37–39]. A cost barrier may also explain the higher enrollment in Za’atri refugee camp, where antenatal services are free and relatively easy to access compared to services out of camp in Jordan. Previous research from Lebanon suggests that unavailability of a reproductive health clinician is also a common barrier to receiving ANC [40]. The observation from our assessments that some women evidently buy iron-folate supplements but do not go for ANC visits may support this finding.

While these data on antenatal care provide some indication that access to reproductive care could be improved, we caution against over interpretation of these findings. Several studies have reported higher utilization of ANC services than we document. In Lebanon, a 2012 clinic based assessment found that among Syrian refugees, 73 % of pregnant women reported at least one antenatal care visit [40]. A non-random, convenience sample of refugees in Lebanon estimated that 83 % of pregnant refugee women attended at least one ANC visit [41]. In Jordan, a nationally representative surveys in 2014, estimated that 82 % of Syrian refugees had made at least one ANC visit while pregnant [37]. Further analysis to explore whether these differences in coverage estimates are attributable to variations in selection of respondents, or whether they reflect true differences in health seeking behaviors, may be useful for scaling up reproductive health services in the region.

Prevalence of diarrhea in children was assessed in all surveys. Two week prevalence of diarrhea was highest in North Lebanon (33.3 %) and Iraq’s Domiz refugee camp (32.8 %). These data suggest an increase in diarrhea prevalence, compared to that reported among children 0–59 in Syria pre-crisis (8.1 %) or the Iraqi population (14.8 %) [14, 34]. Notably, the prevalence of diarrhea in these settings is also high relative to that reported from other refugee camps, including Dadaab camps in Kenya (13.5 %),Tongorara camp in Zimbabwe (21.4 %), and Mai-Ain camp in Ethiopia (29.7 %) [42–44].

This high prevalence is particularly notable given reported access to improved sanitation and safe water. In Lebanon and out of camp in Jordan, nearly all refugees had access to a private toilet; fewer than 2 % of refugees in out of camp settings used public toilets. Even in Za’atri camp, less than a third of households reported using public toilets. For comparison, a global review of 90 refugee camps in 2005 found that on average latrines were shared by 27 people (range 5 to 1124 persons per latrine) [45]. Global analysis have shown an association between prevalence of diarrhea and shared sanitation facilities [46]. For out of camp refugees, the two most common primary sources of drinking water were running water and bottled water. Running water is an improved source of water as classified by the WHO, as is bottled water when another source of water is available for cooking and personal hygiene [47]. In Za’atri, the primary source of water was water tankers, an unimproved source of drinking water [47].

In all settings, reported prevalence of diarrhea was higher among children 0–23 months than children 24–59 months of age. In South and Bekaa regions of Lebanon, prevalence among younger children was more than twice that of older children. The finding of higher diarrhea prevalence among younger children is consistent with previous literature. A 2012 systematic review of data from 72 studies concluded that while rates varied by country, in all regions incidence rates of diarrhea were lower among children aged 24–59 months than among children aged 0–23 months [48]. Data from a case-control study of rotavirus among refugees in Jordan, similarly found rotavirus prevalence was 2.4 times higher in children less than 24 months of age [49].

Current breastfeeding was assessed as a measure of infant and young child feeding practices. Among children under 6 months of age, current breastfeeding was lowest in Domiz refugee camp in Iraq. In Domiz, as well as Za’atri, we document a drop-off in current breastfeeding during the second year of life (12–23 months), such that the proportion breastfeeding in both camps is notably lower than that reported from out of camp refugees in Jordan and Lebanon. These differences may be related to pre-conflict factors, such as the place of origin in Syria and related cultural practices, or may be related to differences in the conditions in the camp that affect breastfeeding practices.

Results reported in this manuscript are subject to several limitations. First, these surveys were initially designed in each country to best inform ongoing emergency response activities. As a result, different survey instruments were used in each country. Results presented here are derived from questions that were reasonably similar across the settings. Standardization of additional questions, particularly questions related to water, sanitation and immunization would have allowed for more comparisons. Second, the assessment in Iraq used a different sampling method than either Jordan or Lebanon assessments. The Iraq survey was designed to be representative of households with children under 5 years of age, not of all households. This methodology did not allow for comparison on several household level indicators with other surveys. Third, self-reported indicators, such as feeding practices, diarrhea morbidity and access to antenatal care, may be subject to recall bias. Finally, no surveys were organized in Egypt or Turkey. Around the time of our surveys, approximately 23 % of Syrian refugees were hosted in Turkey and 6 % were hosted in Egypt. [4] Additionally, while the majority of Iraqi refugees have settled in urban areas, no assessments of non-camp refugees in Iraq were conducted [50]. However, given the similarities in the nutritional status of refugees across the settings assessed, we suggest it is likely that acute malnutrition among the refugees in Turkey, Egypt and non-camp Iraq is similar to those included in our assessments.

## Conclusions

Taken together, the seven surveys of Syrian refugees displaced to Iraq, Jordan, and Lebanon suggest that acute malnutrition was not a significant problem, however refugees from Syria, particularly those in Za’atri refugee camp, experienced elevated prevalence of anemia. In addition to these key findings, we highlight several additional indicators showing similar prevalence across settings and that are important triggers for public health action— low coverage of antenatal care relative to pre-conflict; high prevalence of diarrhea particularly among younger children 0–23 months of age; and low prevalence of continued breastfeeding after 12 months of age.

Given that high prevalence of acute malnutrition is commonly associated with complex humanitarian emergencies, the initial focus of nutrition programming for Syrian refugees focused on treatment of acute malnutrition. However, the findings of these surveys suggest that a drive to scale up treatment of acute malnutrition may not always be the best allocation of scarce resources. While quality treatment of malnourished children at existing health centers remains a priority, these data encouraged a shift in focus of response activities.

Based on the results of these surveys, nutrition partners on the ground prioritized interventions to address micronutrient deficiencies through dietary diversity advocacy and support of food fortification. In Za’atri camp, UN partners transitioned from ration distributions to cash vouchers, a change which allows refugees to shop in markets which offered a wider variety of food, including fresh produce and meat, than that included in the rations. Promotion of appropriate IYCF practices were prioritized to encourage breastfeeding and appropriate introduction of complementary foods. Nutrition partners also recommended inter-sectoral collaboration to promote quality water and sanitation. Given little indication of a quick resolution to the conflict, it was recommended to continue monitoring the situation through periodic nutrition surveys and analysis of facility based surveillance.

## Abbreviations

DHS, Demographic Health Survey; Hb, Hemoglobin; IYCF, Infant and young child feeding; MICS, Multiple Indicator Cluster Survey; SFHS, Syrian Family Health Survey; UNHCR, United Nations High Commissioner for Refugees; UNICEF, United Nations Children’s Fund; WHO, World Health Organization

## Additional file

Additional file 1: Parameters for calculating sample size in Jordan and Lebanon surveys of Syrian refugees, 2013–2014 (DOCX 13 kb)
