# Supplementary material for: Nutritional situation among Syrian refugees hosted in Iraq, Jordan, and Lebanon: cross sectional surveys
Source: Confl Health. 2016 Nov 16;10:26. doi: 10.1186/s13031-016-0093-6 (PMC5111203; doi:10.1186/s13031-016-0093-6)
Supplement: Additional file 1: — Parameters for calculating sample size in Jordan and Lebanon surveys of Syrian refugees, 2013–2014 (DOCX 13 kb) [file 13031_2016_93_MOESM1_ESM.docx]

Additional File 1. Parameters for calculating sample size in Jordan and Lebanon surveys of Syrian refugees, 2013-2014

|  | Jordan 2014 | | Lebanon 2013 | | | |
| --- | --- | --- | --- | --- | --- | --- |
|  | Za’atri  (Refugee Camp) | National  (Outside Camp) | North | South | Beirut | Bekaa |
| Estimated prevalence | 5.8% | 5.1% | 4.4% | 4.4% | 4.4% | 4.4% |
| Required precision | ±3.0% | ±3.0% | ±3.5% | ±3.5% | ±3.5% | ±3.5% |
| Design Effect | 1.1 | 1.9 | 1.44 | 1.44 | 1.44 | 1.44 |
| Percent of children under 5 | 19% | 19% | 19% | 20.9% | 18.9% | 20.7% |
| Average household size | 5.0 | 5.0 | 4 | 4.6 | 4.2 | 4.8 |
| Expected non-response rate | 3% | 3% | 10% | 10% | 10% | 10% |
| Sample size – children aged 6-59 months | 279 | 427 | 207 | 207 | 207 | 207 |
| Sample size – households | 337 | 515 | 340 | 270 | 330 | 260 |
